# 

**Published:** 1892-11-05

**Authors:** 


Notices of Births, Deaths, and Marriages, are inserted in this column at a charge of 2s. 6d. for an announcement not exceeding four lines.

### NOTICE TO CORRESPONDENTS.

All MS., Letters, Books for Review, and other matters intended for the Editor should be addressed THE EDITOR, THE LODGE, PORCHESTER SQUARE, LONDON, W.

The Editor cannot undertake to return rejected M.S., even when accompanied by stamped directed envelope.

**Notice to Advertisers and Subscribers.**

All Advertisements, Orders for Copies of THE HOSPITAL, and Business Communications should be addressed to The Publisher, *not to the Editor*, THE HOSPITAL, 140, Strand, W.C.

The Cost of Subscription, post free, is as follows:—Per week, 2½d.; per quarter, 2s. 6d.; per half-year, 5s.; per annum, 8s. 6d. Foreign:—Per annum, 10s. 8d.; payable, in all cases, in advance.

**"Burdett's Hospital Annual," 1891—1892.**

Third year of publication. 600 pp. Boards, gilt lettering. A most complete guide to British and Colonial Hospitals, Dispensaries, Nursing and Convalescent Institutions, and Asylums. Price 3s. 6d. Published by THE SCIENTIFIC PRESS (Limited), 140, Strand, W.C.

**NOTICE.—The Index for Vol. XII. (ending September, 1892) is on sale at the Office of this Journal, price 2½d., post free.**

### Scraps and Gleanings.

Catherine Sweeney, of Ballycallen, County Kilkenny, has died at the age of 110 years.

The Marquis of Bute has sent a donation of £25 towards the new wards of the Luton Cottage Hospital.

The Hospital Saturday collections this year at Colchester amounted to £151 12s. 7½d. At Swindon to £44 8s. 11¾d.

The Duchess of Teck has promised to open a bazaar at Enfield on November 19th in aid of the Cottage Hospital.

This year the collections on Hospital Sunday at Leominster were given to the fund for starting a cottage hospital in that town.

The Hospital Saturday collections from the workpeople of Leicester towards the infirmary amounted this year to £1,844 11s. 5d.

The new wing of the Ashton Infirmary is the gift of Mr. Kershaw, J.P., of Ashton-under-Lyne, and is to be used as a Children's Hospital.

The sad death of a woman named Gower is reported from Sellirgde, Kent. While sitting near the fire she fell asleep, her clothing became ignited, and she was burned to death.

The Wombwell Hospital report shows that last year 2 013 out and 363 in patients were treated. On Hospital Sunday the sum of £35 19s. 3d. was collected in aid of the institution.

The late General Doria, who was up to the time of his death a member of the Board of Management of the Chelsea Hospital for Women, has bequeathed £1,000 to its funds.

The site for the General Hospital to be erected at Kettering has been given by the Duke of Buccleuch. The amount of donations and subscriptions already promised amount to £3,987 16s.

Mrs. Brassey and family, as a memorial to the late Mr. H. A. Brassey, have added a wing of four dwellings, endowed with £100 a year, to the Hospital of the Holy Trinity, at Aylesford.

The site for the new convalescent home for Leicestershire is situated about three miles from Loughborough. The building is to cost about £5,000. Towards this amount £3,500 has been promised.

The enlargement of the chapel of the Hospital for Consumption, Brompton, was recently consecrated by the Bishop of London. The additions have been made to commemorate the jubilee of the hospital.

Sir John Bridge has granted Police-constable Alexander Rowland £5 from the Bow Street poor-box, in recognition of his gallant conduct in attempting to save a man who was recently drowned in the Thames at Battersea.

The Hope Hospital for Infectious Diseases, which was recently opened at Warrington, contains two large wards, one for men, and the other for women; in the centre of the two is a specially constructed apartment for the three nurses.

At the annual meeting of the Lowestoft Hospital it was decided to open a subscription list for the proposed alterations and additions to the Hospital, which are so greatly needed. The number of in patients treated was 322, out-patients 1,198.

It is hoped that the City Council will be induced to grant a sum of money as well as a site for the proposed new Northern Hospital. For a long time past the present building has been found quite inadequate, and a new one has been imperatively demanded.

Lord and Lady Wantage have offered, as a free gift to the Northampton County Council, Abington Abbey and twenty acres of land, for use as a People's Park. The grounds are well planted with lovely trees, and the Abbey is a very old and picturesque building.

At the annual meeting of the Governors of the Falmouth Dispensary it was decided to try and make arrangements for the rental of the present Cottage Hospital temporarily, until further steps could be taken in regard to building a new and larger hospital.

The Prince of Wales has promised to lay the foundation-stone of the Clarence Memorial Wing of St. Mary's Hospital, Paddington, on December 17th. A presentation of purses in aid of the Clarence Memorial Fund will take place, each purse to contain a sum of not less than five guineas.

It is proposed to commence building at once the administrative block of the new Blackpool Hospital, leaving the two wings, as well as the back portion of the building, until the funds have been raised to defray the cost. A large bazaar is announced to take place next year in aid of the building fund.

The late Dr. Beaney, of Melbourne, has bequeathed £10,000 to the Mayor and Corporation of Canterbury, his native city, to buy a suitable piece of land, whereon to erect a free library and reading-room for the working classes, the building when erected to be called the Beaney Institute for the Education of Working Men.

The North Birley Joint Hospital for Infectious Diseases just opened is for the use of the inhabitants of the Local Board Districts of Cleckheaton, Hunsworth, North Bierley and Tong. The buildings comprise an administrative block, a ward pavilion with twelve beds and six cots, and an isolation block with ten beds and four cots.

In Sunderland district there were 511 births and 570 deaths in the year. The death-rate was equal to 38 per 1,000, whilst the health returns for the kingdom show that the death-rate in Sunderland district should not exceed 19 per 1,000. It is time that steps were taken to secure Parliamentary powers for dealing with the worst and most unsanitary blocks.

The Norfolk and Norwich Hospital is in need of funds. Last year the ordinary income was only £7,326, whereas the ordinary expenditure amounted to £8,871, showing a deficiency of no less than £1,545. Unless an additional thousand pounds a year is subscribed, the board of management will be obliged to close one or more wards, and in order to avert it, prompt steps should be taken at once to raise the required sum.

The new Workhouse Infirmary, Hull, to accommodate 100 patients, male and female, has been built by order of the Local Government Board at a cost of about £6,000. The building is two storied. The administrative block occupies the centre, with the men's wards to the north, and the women's to the south. Special provisions have been made in case of fire. The plans were drawn up by Mr. H. Brownlow Thompson.

### THE STUDENT OF MEDICINE.

### APPOINTMENTS.

Allan, Francis J., M.D., D.P.H., appointed Lecturer in Sanitary Science, King's College, London. Bassano, T. M., M.B., C.M.Edin., L.R.C.P. and S.Edin., appointed House Surgeon to the Huntingdon County Hospital. Body, H. M., M.R.C.S.Eng., re-appointed Medical Officer of Health for Crediton Urban Sanitary District. Burns, R. J., L.R.C.P.Lond., M.R.C.S., appointed Police Surgeon for the Borough of Sunderland. Burton, Dr., re-appointed Medical Officer of Health for the Greenford Rural Sanitary Authority. Conway, A., M.R.C.S., L.R.C.P., appointed Medical Officer for the Kingwinford No. 2 District of the Stourbridge Union. Coupland, Sidney, M.D., F.R.C.P.Lond., appointed Examiner in the Practice of Medicine and Pathology at the University of Aberdeen. Craven, R. M., L.R.C.P.Edin., M.R.C.S.Eng., re-appointed Medical Officer of Health for the Westmoreland Combined Districts. Derry, Bartholomew G., L.R.C.P.Lond., M.R.C.S.Eng., re-appointed Medical Officer of Health for the Bodmin Urban District. Dryland, Leslie W., M.R.C.S., L.R.C.P., appointed Assistant House Surgeon to the Northampton General Infirmary. Edmond, George, M., M.D.Aberd., appointed Examiner in Surgery and Midwifery at the University of Aberdeen. Elliman, Arthur C., L.R.C.P.Lond., M.R.C.S., appointed House Surgeon to the Kent and Canterbury Hospital. Evans, William, L.R.C.P.Edin., M.R.C.S., re-appointed Medical Officer of Health Anglesey Rural District. Fussell, Edward Francis, M.B.Aberd., M.R.C.P.Lond., re-appointed Medical Officer of Health for East Sussex. Galloway, James, M.D., appointed Examiner in Botany and Materia Medica at the University of Aberdeen. Gilpin, Frank, M.R.C.S.Eng., re-appointed Medical Officer of Health to the Stratford-upon-Avon Town Council. Hill, Alfred, M.D.Aberd., M.R.C.S., appointed Examiner in Chemistry and Medical Jurisprudence at the University of Aberdeen. Hoskins, E., M.R.C.S., re-appointed Medical Officer for the Little Eaton Sanitary District of the Shardlow Union. Hoskins, William, M.B., C.M.Aberd., appointed Medical Officer for the Nazeing District of the Epping Union. Jones, Dr., re-appointed Medical Officer of Health for the Borough of Ruthin. Marriott, H., M.B., M.R.C.S., appointed Surgeon to the Stockport Infirmary. Mason, George A., M.A., M.B., B.C.Cantab, appointed House Physician to the Great Northern Central Hospital. Mercer, W. B., B.A., M.B., B.C.Cantab, M.R.C.S., L.R.C.P., appointed House Physician to the Royal Hospital for Diseases of the Chest, City Road, E.C. Morse, E., L.R.C.P., L.R.C.S.Edin., re-appointed Medical Officer of Health for Torrington. Mullally, W. T., M.D., Dip. San.Science R.C.P.I., appointed Health Officer to the Council of the Shire of Ballarat, Victoria. Norris, G. R., M.R.C.S.Eng., re-appointed Medical Officer of the No. 2 District of the Dulverton Union. Norton, John, M.D., D.P.H., appointed Medical Officer of Health to St. Peter's Close, Westminster. Ortlepp, A. J., M.R.C.S., L.R.C.P.Lond., appointed House Surgeon to the Female Lock Hospital, Harrow. Pearse, Francis J., M.R.C.S., appointed Divisional Surgeon to the A Division of the Metropolitan Police, and Medical Officer to the United Westminster Almshouses. Prankerd, Herbert Peter, M.B., C.M.Edin., appointed Medical Officer for the No. 2 District of the Southampton Incorporation. Pullin, T. H. S., M.D. St.And., F.R.C.S.Edin., re-appointed Medical Officer of Health to the Sidmouth Local Board. Radford, William J., L.R.C.P. Lond., M.R.C.S.Eng., appointed Senior House Surgeon to the Poplar Hospital for Accidents. Ralph, Hugh, M.R.C.S., L.R.C.P., appointed Junior Resident Medical Officer to the Evelina Hospital for Sick Children, Southwark Bridge Road. Rennet, David, M.B., C.M., D.P.H., appointed Assistant Professor of Medical Jurisprudence in the University of Aberdeen. Robertson, J. S., M.R.C.S., appointed Medical Officer for the Bloxham Sanitary District of the Banbury Union. Snadden, James, M.B., C.M.Edin., re-appointed Medical Officer for the No. 4 District of the Wortley Union. Stewart, George N., M.D., appointed Examiner in Natural History and Institutes of Medicine at the University of Aberdeen. Swallow, F. McDonald, L.R.C.P., L.R.C.S.Edin., appointed Medical Officer of Health for the Penistone Rural Sanitary District of the Penistone Union. Trotman, Frank, M.B., L.R.C.P., M.R.C.S., appointed Medical Officer of the Bristol Town Council. Wacher, Frank, M.R.C.S., re-appointed Medical Officer of Health for the Canterbury Urban Sanitary District. Willcocks, Arthur Durant, M.R.C.S.Eng.,

### The Hospital Advertiser.

### MEDICAL APPOINTMENTS.

**THE HOSPITAL FOR SICK CHILDREN,**

THE STUDENT OF MEDICINE—(continued).

appointed Medical Officer for the Taunton Union Workhouse. Wilson, G., M.D., D.P.H., appointed Medical Officer of Health of the Meriden, Rugby, Solihull, Southam, and Warwick Unions, and the Kenilworth, Rugby, and Warwick Urban Sanitary Districts. Windle, Bertram C. A., M.D., appointed Examiner in Anatomy at the University of Aberdeen. Woollett, Sidney W., M.R.C.S., appointed Medical Officer of No. 8 District of the Blything Union. Wright, H., L.R.C.P., L.M.Edin., M.R.C.S., re-appointed Medical Officer of Health for the Gainsborough Rural Sanitary District. May, Wm. Page, M.D., B.Sc.Lond., M.R.C.S., M.R.C.P., has been appointed Pathologist vice Dr. Chaplin promoted Assistant Physician.

